# A mechanism-based proof of concept study on the effects of duloxetine in patients with painful knee osteoarthritis

**DOI:** 10.1186/s13063-021-05941-y

**Published:** 2021-12-27

**Authors:** Nadia Ammitzbøll, Lars Arendt-Nielsen, Davide Bertoli, Christina Brock, Anne Estrup Olesen, Andreas Kappel, Asbjørn Mohr Drewes, Kristian Kjær Petersen

**Affiliations:** 1 Department of Gastroenterology and Hepatology, Aalborg University Hospital, Aalborg, Denmark; 2 Center for Neuroplasticity and Pain, SMI, Department of Health Science and Technology, Faculty of Medicine, Aalborg University, Aalborg, Denmark; 3 Center for Neuroplasticity and Pain, Department of Health Science and Technology, Faculty of Medicine, Aalborg University, Aalborg, Denmark; 4 Department of Clinical Medicine, Faculty of Medicine, Aalborg University, Aalborg, Denmark; 5 Department of Clinical Pharmacology, Aalborg University Hospital, Aalborg, Denmark; 6 Department of Orthopaedics, Aalborg University Hospital, Aalborg, Denmark

**Keywords:** Quantitative sensory testing, Chronic pain, Osteoarthritis, Duloxetine

## Abstract

**Background:**

The global burden of osteoarthritis (OA) is steadily increasing due to demographic and lifestyle changes. The nervous system can undergo peripheral and central neuroplastic changes (sensitization) in patients with OA impacting the options to manage the pain adequately. As a result of sensitization, patients with OA show lower pressure pain thresholds (PPTs), facilitated temporal summation of pain (TSP), and impaired conditioned pain modulation (CPM). As traditional analgesics (acetaminophen and non-steroidal anti-inflammatory drugs) are not recommended for long-term use in OA, more fundamental knowledge related to other possible management regimes are needed. Duloxetine is a serotonin-noradrenalin reuptake inhibitor, and analgesic effects are documented in patients with OA although the underlying fundamental mechanisms remain unclear. The descending pain inhibitory control system is believed to be dependent on serotonin and noradrenalin. We hypothesized that the analgesic effect of duloxetine could act through these pathways and consequently indirectly reduce pain and sensitization. The aim of this mechanistic study is to investigate if PPTs, TSP, CPM, and clinical pain parameters are modulated by duloxetine.

**Methods:**

This proof of concept study is a randomized, placebo-controlled, double-blinded, crossover trial, which compares PPTs, TSP, and CPM before and after 18 weeks of duloxetine and placebo in forty patients with knee OA. The intervention periods include a titration period (2 weeks), treatment period (60 mg daily for 14 weeks), and a discontinuation period (2 weeks). Intervention periods are separated by 2 weeks.

**Discussion:**

Duloxetine is recommended for the treatment of chronic pain, but the underlying mechanisms of the analgesic effects are currently unknown. This study will investigate if duloxetine can modify central pain mechanisms and thereby provide insights into the underlying mechanisms of the analgesic effect.

**Trial registration:**

ClinicalTrials.govNCT04224584. Registered on January 6, 2020. EudraCT 2019-003437-42. Registered on October 22, 2019. The North Denmark Region Committee on Health Research Ethics N-20190055. Registered on October 31, 2019.

## Background

Osteoarthritis (OA) is highly prevalent in the elderly population, and the prevalence of OA increases with increasing age and obesity, and thereby the prevalence is expected to rise [12]. Duloxetine is a serotonin and norepinephrine reuptake inhibitor and is currently approved for patients with knee OA and recommended by the Osteoarthritis Research Society International (OARSI) for use in patients with knee OA and widespread pain and/or depression [10]. Six randomized controlled trials have been conducted on the efficacy of duloxetine in patients with OA, which have all demonstrated analgesic effects favoring duloxetine over placebo [17] with an effect size of 0.55 [42]. The underlying mechanisms of the analgesic effects in OA remain largely unknown. Yarnitsky et al. 2012 [61] administered duloxetine to patients with diabetic neuropathies and found a stronger analgesic effect in patients having impaired conditioned pain modulation (CPM) prior to treatment, indicating that patients with a neuropathic pain-like component might respond better to duloxetine.

Recent reviews have concluded that a neuropathic pain-like component is present in subpopulations of patients with OA [20, 33, 38]. Lower pressure pain thresholds (PPTs) at the knee and remotely from the knee are found in patients with severe knee OA compared with healthy subjects, indicating localized and widespread pressure hyperalgesia [3, 6]. Temporal summation of pain (TSP) is a proxy for central nervous system excitability [50]. TSP is facilitated in severe knee OA patients compared with healthy subjects [4, 6] and has been found to be a predictor of chronic postoperative pain [32, 43, 47]. TSP has also been found to be a predictor of limited analgesic effect of non-steroidal anti-inflammatory drugs (NSAIDs) [46] in OA. CPM is a proxy for descending pain inhibitory control [60], is impaired in severe patients with OA [6] and impaired CPM has been associated with higher levels chronic postoperative pain [34, 57] and poor analgesic effects to NSAIDs effects in patients with OA [21, 48].

Limited evidence is currently available on how to treat sensitization in OA patients, but emerging indications suggest that duloxetine might be such a treatment [61].

It is expected that patients with OA pain may benefit from the duloxetine therapy as a central modulator of pain processing [6].

This proof-of-concept randomized placebo-controlled double-blinded crossover trial aimed to investigate the effect of 18 weeks of duloxetine on mechanistic pain profiles when comparing to placebo in patients with severe OA. The primary objective was to assess the treatment effect on mechanistic pain profiles (PPTs, TSP and CPM) when comparing duloxetine to placebo.

## Methods and Analysis

### Study design

**Fig. 1:**
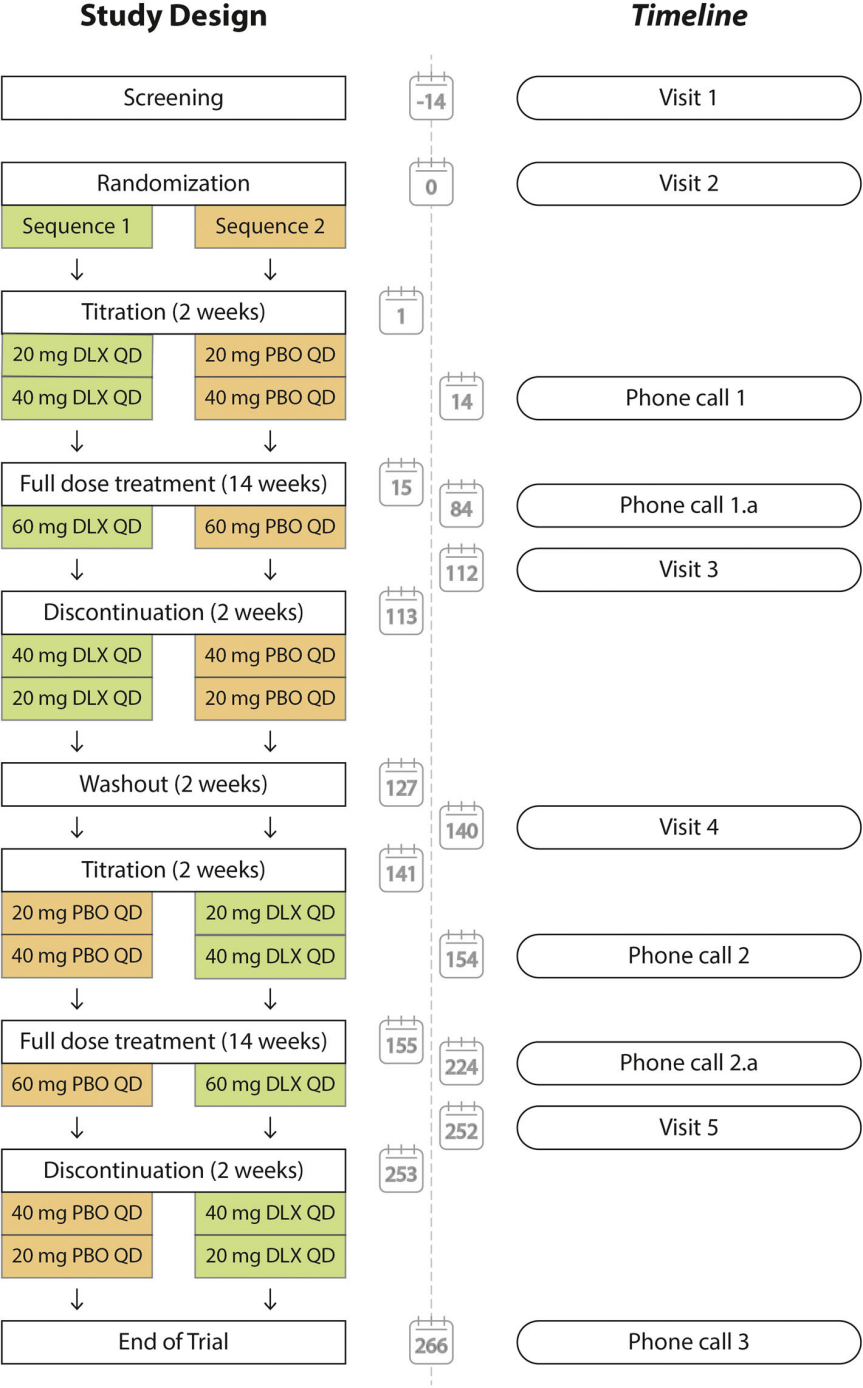
Study design and timeline. Patients are randomized to either duloxetine followed by placebo (sequence 1) or placebo followed by duloxetine (sequence 2). Approximate dates for each study event (visits or phone calls) are highlighted in the timeline. Abbreviations: DLX, duloxetine; PBO, placebo; QD, daily dose

This proof of concept study is a randomized, placebo-controlled, double-blinded, crossover trial, which compares PPTs, TSP, and CPM before and after 18 weeks of duloxetine and placebo. Forty patients will be randomized to one of two equally sized sequences: (1) duloxetine followed by placebo or (2) placebo followed by duloxetine (Fig. 1). Patients will be screened for inclusion (visit 1) and assessed at baseline (visit 2 and visit 4) and after the intervention (visit 3 and 5).

**Fig. 2:**
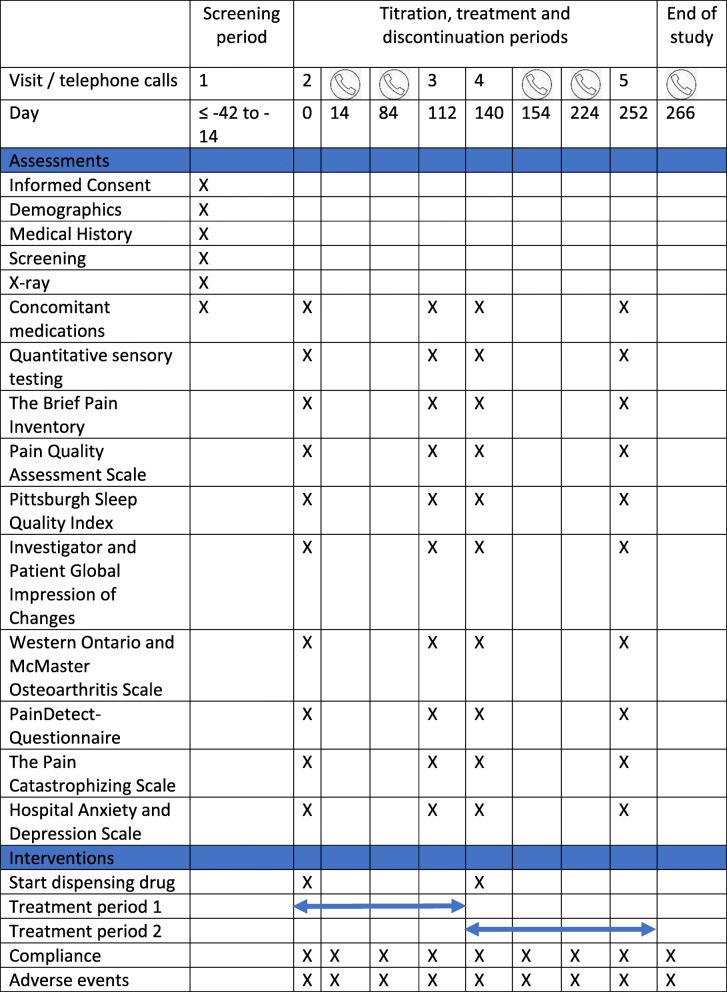
A Standard Protocol Items: Recommendations for Interventional Trials (SPIRIT) schematic diagram of the interventions and assessments in the trial

Randomization is conducted before baseline measurements at visit 2. During the visits, the patients will be assessed using quantitative sensory testing (QST) and questionnaires. In addition, patients are continuously asked to rate adverse events using a daily diary and the diary is assessed at each visit. Please find a complete outline of the intervention periods and assessments in Fig. 2.

### Study Population and implementation

Patients are recruited through Synexus, Aalborg, Denmark (a contracting research organization). Women and men, 40–75 years of ages, with OA of the knee, who are eligible to be included in the study, agree to participate and fill in an informed consent, will be included. Patients fulfilling the inclusion criteria will be invited to a screening visit (visit 1) where a physical and psychological examination (for evaluation of suicidal risk) will be conducted by a medical doctor. The screening includes x-ray of the knee, screening of medical records, and screening of the eligibility criteria and will be handled by Synexus, Aalborg, Denmark.

Patients will be asked to discontinue the use of all analgesic pain medication (including NSAIDs) during the entire trial and any need of rescue medication (paracetamol) must be noted in the diary.

Patients who are eligible according to the inclusion and exclusion criteria will be invited to visit 2 where they will be included in the study and randomized.

Following visits 1, the entire trial including all patient communication and randomization will be handled by MechSense, Department of Gastroenterology and Hepatology, Aalborg University Hospital, Denmark.

The allocation sequence will be generated by the Hospital Pharmacy at Aarhus University Hospital, Aarhus, Denmark, and all study personal (except dedicated staff at the hospital pharmacy) and patients are blinded to the allocation sequences.

### Inclusion criteria and exclusion criteria

#### Inclusion criteria

Patients diagnosed with unilateral or bilateral OA of the knee according to the American College of Rheumatology (ACR) criteria based on clinical and radiographic evidence [1] will be recruited. In addition, patients must provide written informed consent and abide by the study restrictions. The patients must have a Kellgren and Lawrence grade of I, II, or III at the index knee. Worst pain within the last 24 h must be 5.0 cm to 10.0 cm (assessed on a 0–10-cm VAS scale anchored at 0 cm: no pain and 10 cm: worst pain imaginable) prior to enrolment. patients must agree to maintain the same activity level throughout the course of the study.

#### Exclusion criteria

Enrolment is restricted to people aged 40 years or older because knee pain in younger patients is mainly due to trauma rather than to naturally occurring OA. Patients are screened for suicidal risk using the Columbia-Suicide Severity Rating Scale [49] and patients at risk are excluded to ensure patient welfare. Interactions with other drugs are essentially unknown. Therefore, patients having specific medical conditions other than OA or taking specific medications other than the allowed are excluded because of the potential interaction of those conditions or medications with known or potential effects on duloxetine. Patients taking certain psychoactive medications, abusing drugs or alcohol, or having other dependencies are excluded because of the potential confounding factors of these medications/substances on the results.

Since the purpose of the study is to evaluate the mode of action of duloxetine in treating the pain of osteoarthritis of the knee, patients with other conditions that might produce pain in or alter the perception of the osteoarthritic pain in the index knee are excluded to avoid confounding the assessment of the primary outcome measure.

### Removal of patients

Patients will be informed that they are free to discontinue from the study at any time and for any reason. The investigator may remove a patient from the study in case of any clinical adverse event, laboratory abnormality, or current illness, which in the opinion of the investigator indicates that continued participation in the study is not in the best interest of the patient. Patients may be discontinued due to a change in compliance with inclusion/exclusion criteria that is clinically relevant and affects patient safety, occurrence of adverse events, or ingestion of non-permitted concomitant medication that might affect the patient safety or study assessments/objectives.

In case of premature discontinuation of study participation, efforts will be made to conduct all end-of-study assessments. All patients who prematurely discontinue will be followed for ongoing and newly occurring adverse events.

### Interventions

Study interventions are duloxetine treatment and placebo treatment. Patients are block-randomized (four patients at a time) to either sequence 1 (e.g., duloxetine followed by placebo) or sequence 2 (e.g., placebo followed by duloxetine). All patients will be administered an equal number of capsules containing duloxetine or placebo. Patients are thoroughly instructed on the most common adverse events and on how to report these.

Treatment periods include 2-week titration periods (week 1 (7 days): 20 mg/daily, week 2 (7 days): 40 mg/daily), a 14-week full treatment period (week 3–16 (70 days): 60 mg/daily) followed by a 2-week discontinuation period (week 17 (7 days): 40 mg/daily, week 18 (7 days): 20 mg/daily). The treatment periods are separated by at least two weeks.

The patients are instructed to take one capsule of study drug orally with approximately 200 mL of water at room temperature after breakfast in the morning of each dosing day.

### Blinding

The study drug is encapsulated in a gelatin capsule (DBCaps® from Capsugel, size: AAEL, color HPMC Swedish Orange Opaque) with identical size, color, and weight to ensure blinding.

The study drug is produced and labeled by the Hospital Pharmacy at Aarhus University Hospital. The blinding and randomization procedures are conducted by the Hospital Pharmacy at Aarhus University Hospital, Aarhus, Denmark. Patients, study personnel, and study management will be blinded until the end of the trial. Unblinding will take place if the patient’s safety is at risk to ensure allocation concealment.

### Adverse events

The principal investigator is responsible for monitoring the safety of the patients who have entered this study and for alerting Sponsor of any serious adverse event. Adverse events will be monitored from the time when the patient has signed the informed consent form and until two weeks after the last drug administration (end of study).

### Ancillary and post-trial care

If the participant should become ill or suffer injury as a direct result of the study drug or experimental procedure, he/she must inform the clinical investigator who will arrange for the proper treatment and help with further developments. This also applies if the participant will complain about the treatment.

The patients are covered by Aalborg University Hospital Patient Insurance.

### Outcomes

#### Primary outcome

The primary outcome parameters are the effects of duloxetine on experimental pain stimulations (pressure pain thresholds, cuff pain detection and tolerance thresholds, temporal summation of pain, and conditioned pain modulation).

##### Pressure pain threshold

Quantitative sensory testing of joint pain will be determined by assessing PPTs from three knee joint locations at the most painful side (the index knee). A hand-held pressure algometer (Somedic) will be used for measuring PPTs. The probe (1 cm^2^) will be placed perpendicularly to the skin and pressure will be applied until the patient defines the pressure as pain and presses a button. Spreading sensitization will be assessed by PPTs from tibialis anterior of the most painful knee and from the extensor brachii radialis muscle on the ipsilateral side. PPTs will be measured twice on each site and the mean of the two measurements will be used in the statistical analysis.

##### Cuff pain detection and tolerance threshold

Deep tissue pain sensitivity will be evaluated by cuff pressure stimuli using a computer-controlled cuff algometer (Cortex Technology and Aalborg University, Denmark) including a 13-cm wide tourniquet cuff (VBM, Sulz, Germany) and an electronic VAS (Aalborg University, Denmark) for recording of the pain intensity. The cuff will be placed at the level of the head of the gastrocnemius muscle of the lower leg at the index knee. The electronic continuous VAS (sliding resistor) is 10 cm long and sampled at 10 Hz; 0 cm indicates “no pain” and 10 cm indicates “maximum pain.” Cuff algometry is a reliable assessment for PPTs, TSP, and CPM [24, 30] and has often been utilized in studies with OA patients [32, 44, 46, 48].

The pressure of the cuff is increased by 1 kPa/s and the patient will be instructed to rate the pain intensity continuously on the electronic VAS until the tolerance level is reached. The patient will be instructed to press a stop button at this point. The pressure pain detection threshold (cPDT) is defined as the pressure at which the VAS score exceeds 1 cm. The pain tolerance threshold (cPTT) is defined when the patient presses the stop button. The measurements will be conducted once on both the ipsilateral and contralateral lower leg to the most OA-affected knee.

##### Temporal summation of pain

Ten short-lasting stimuli (1 s each) at the level of the cPTT will be given at the lower leg with a 1 s break between stimuli. The participants are instructed to continuously rate the pain intensity of the sequential stimuli using the electronic VAS and not return to zero during the breaks. For each cuff stimulus, a VAS score will be extracted.

##### Conditioned pain modulation

The CPM magnitude is assessed as the absolute changes in cPDT with and without a cuff conditioning stimulus. A conditioning stimulus will be applied to the contralateral lower leg and the cPDT will be assessed on the ipsilateral lower leg as described above. The conditioning stimulus is applied as a constant stimulus with an intensity of 70% of the pain tolerance level on the contralateral leg.

#### Secondary outcome

Secondary outcomes are assessed to evaluate the effect of duloxetine and placebo on clinical pain intensities and quality, function of the knee, quality of sleep, pain catastrophizing, anxiety, and depression, as described below.

Patients will rate the pain severity for worst pain during the day and pain severity at night on a 10 cm VAS scale. Pain severity will be recorded on a daily basis by the patients in the patient diary.

##### Pain Quality Assessment Scale

This measure begins with an introduction stating that people experience pain sensations differently and explaining how pain unpleasantness differs from pain intensity. After the introduction, the patients are asked to rate the severity of each of the 20 pain domains using 0 to 10 numerical rating scales in which 0 = ”no pain” or “no (sensation/item)” and 10 = “the most (descriptor) pain sensation imaginable.” As mentioned above, the pain domains assessed include two global domains (pain intensity and unpleasantness), two spatial domains (deep and surface), and 16 quality domains (sharp, hot, dull, cold, sensitive, tender, itchy, shooting, numb, electrical, tingling, cramping, radiating, throbbing, aching, and heavy).

##### Pittsburgh Sleep Quality Index

The Pittsburgh Sleep Quality Index is a self-rating questionnaire resulting in a global score between 0 and 21. The questionnaire consists of seven sub-scores (sleep quality, sleep onset latency, sleep duration, sleep efficiency, sleep disturbances, use of sleeping medication, and daytime dysfunction) [16].

##### The Brief Pain Inventory

The Brief Pain Inventory [18, 55] (severity and interference scores) is a self-reported scale that measures the severity of pain and the interference of pain on function. The scores range from 0 (no pain) to 10 (pain as severe as you can imagine). Four questions assess the worst pain, least pain, and average pain in the past 24 h, and the pain right now. The interference scores range from 0 (does not interfere) to 10 (completely interferes). Seven questions assess the interference of pain in the past 24 h for general activity, mood, walking ability, normal work, and relations with other people, sleep, and enjoyment of life.

##### Investigator Global Impression of Changes

The Investigator Global Impression of Changes is a questionnaire used by physicians to provide a global impression of change in the patient’s health whether or not it is related to observational study treatment. The Investigator Global Impression of Changes is often used to show that a change in the patient is clinically meaningful. This instrument asks the physician to answer the following question, “Compared to the patient‘s condition at admission to this study, how much has he or she changed?” on a seven-point scale with + 3 equaling very much improved and − 3 equaling very much worse [26]. The Investigator Global Impression of Changes and other global impression questionnaires have a long history of use across a number of therapeutic areas and indications.

##### Patient Global Assessment of Changes

This questionnaire assesses the change in their health status since beginning the study treatment. Like the Investigator Global Impression of Changes, the Patient Global Assessment of Changes has been used frequently in clinical and observational studies across the spectrum of therapeutic areas and products. The Patient Global Assessment of Changes is useful in assessing whether the impact of the study treatment is meaningful enough to give value to the patient [56].

##### Western Ontario and McMaster Osteoarthritis Scale

The Western Ontario and McMaster Osteoarthritis Scale [11] is a patient-rated instrument that measures OA symptoms. The questionnaire contains five pain questions, two stiffness questions, and 17 physical function questions (24 questions total). Each question utilizes a 5-point scale from 0 (none) to 4 (extreme).

##### PainDetect-Questionnaire

The PainDetect Questionnaire has been developed to assess the neuropathic components of a given pain disease [23]. The PainDetect-Questionnaire is a validated, easy to use, screening tool that predicts the likelihood of a neuropathic pain component in chronic pain disorders [23]. It shows higher sensitivity and specificity in comparison with other neuropathic pain screening questionnaires.

##### The Pain Catastrophizing Scale

The Pain Catastrophizing Scale consists of 13 items focusing on thoughts and feelings in connection with pain [54]. The questions are to be rated on a 4-point scale ranging from 0 (not at all) to 3 (very much).

##### Hospital Anxiety and Depression Scale

Anxiety and depression symptoms are assessed using the Hospital Anxiety and Depression Scale (36), which applies a subscale for anxiety and a subscale for depression. The Hospital Anxiety and Depression Scale ranges from 0 to 21: 0 to 7 indicate no symptoms of anxiety/depression, 8 to 10 indicate possible symptoms of anxiety/depression, and 11 to 21 indicate severe symptoms of anxiety/depression [62].

##### Sample size

Wang et al. 2019 [2] demonstrated an effect size of 0.55 when assessing the worst pain within the last 24 h for 10-week treatment of duloxetine compared with placebo in a parallel design with patients with moderate-to-severe OA. It is believed that the effect of duloxetine acts through descending pain pathways and therefore the decrease in pain intensity must be associated with the modulation of pain mechanisms. A sample equation with 85% power and a significant level at 0.05 using a crossover design yields 32 patients. Forty patients will be enrolled to account for potential drop-outs.

### Statistical analysis plan

#### Primary outcomes

Repeated Mixed Models (RMMs) will be used to compare duloxetine to placebo in changes from baseline to treatment for PPTs, cPDT, cPTT, TSP, and CPM. Different variance-covariance structures will be explored using secondary outcomes. Regression analysis using primary and secondary outcomes will be explored to predict the analgesic effect of duloxetine.

#### Secondary outcomes

The secondary efficacy analyses involving duloxetine comparison with placebo will use the secondary outcomes listed above.

The secondary efficacy measures will be analyzed using an RMM approach and potentially covariates will be included. If the RMM analysis shows a significant effect for a variable, contrasts will be constructed for that variable to provide a post hoc pairwise comparison for each time point. Furthermore, on an exploratory basis, pairwise comparisons may be carried out between duloxetine and placebo at each time point.

Analyses focused on responders and non-responders will be conducted for all study parameters with the responder being defined as a minimum reduction in pain from baseline to follow-up after duloxetine intervention of 30% or 50%.

In addition, predictive models using pretreatment parameters will be used to predict the response rate of the duloxetine intervention.

### Ethical considerations

Duloxetine has multiple side effects. Therefore, the patients included in the current study are limited by an extensive exclusion criteria list to ensure that included patients tolerate the study drug. The patients are instructed to inform the study personal of any adverse events. Despite the side effects, duloxetine has been documented to provide analgesic effect to patients who are “centrally sensitized” [61], and the results from the proposed study could act as a proof-of-concept study for new pain treatment of patients with OA and who are severely centrally pain sensitized.

Further, the patients will experience pain during the experimental pain sessions, to which they would normally not be exposed. However, patients will be instructed to push a stop button when the pain becomes intolerable and the test will stop immediately. The patients might experience redness or soreness in the hours following the experimental pain tests, but no long-term effects have been reported by using the methods proposed in this protocol [28, 44–46, 51].

A radiological assessment of the knee (an x-ray) is required to determine the eligibility of OA in the patients. This exposes the patients to radiation, which would normally not occur. The radiation mSv dose for x-ray of the knee will be less than 0.1 mSv, which corresponds to a few days of background radiation and causes a negligible cancer risk. Blood samples will be collected in the study, which may cause bruising of the skin and involve a minimal risk of infection. Trained personnel will conduct the procedures and optimal equipment will be used to limit the risk of x-ray radiation and infections during blood samples to an acceptable level.

The patients enrolled will be asked not to use their current analgesics (up to 4000 mg paracetamol daily is allowed) and to use the study drug only. Paracetamol might not be able to render complete pain relief to the patients enrolled and therefore the patients are likely to feel pain (especially during the placebo phase).

### Trial steering committee

AMD, LAN, and KKP participated in the conception of the study design and procured funding. LAN and KKP act as sponsors for the trial, AMD acts as principal investigator and ensures the safety of the study participants and KKP is coordinating the ongoing trial. The steering committee reviews the progress of the trial and agrees on necessary changes to the protocol.

### Modification of the protocol

Any modifications to the protocol which may impact on the conduct of the study, potential benefit of the patient or may affect patient safety, including changes of study objectives, study design, patient population, sample sizes, study procedures, or significant administrative aspects will require a formal amendment to the protocol. Such amendment will be agreed upon by trial steering committee and approved by the Ethics Committee and the Danish Medicines Agency prior to implementation.

### Data collection and management

All data collected are entered in RedCap (version 8.10.1, Vanderbilt University, TN, USA) and will be transferred to SPSS (version 25.0, SPSS Inc., Chicago, IL, USA) for statistical analysis.

### Audit and monitoring

The trial is monitored by the GCP-unit, Denmark (https://gcp-enhed.dk/), and the monitor has been appointed by the sponsor. The Danish Medicines Agency and The North Denmark Region Committee on Health Research Ethics may conduct audits which are independent of the sponsor.

### Confidentiality

All datasets will be fully patient-anonymized.

### Dissemination policy

The trial steering committee is responsible for publishing the results regardless of the outcome. The trial steering committee will discuss potential new publications based on the collected material. Authorship is rewarded based on the guidelines from the International Committee of Medical Journal Editors [31].

## Discussion

The increasing prevalence of obesity and the increasing age of the world population are two factors which may be linked to the increasing prevalence of OA [12]. Standard OA treatments such as exercise-based therapy, NSAIDs, and total joint arthroplasty are efficient in many patients with OA. However, it seems evident that a subset of patients does not gain sufficient pain relief from these treatments [13, 14, 53]. Long-term pharmacological treatment of OA pain is problematic due to side effects and opioids are not advised for treatment of OA pain [10], why new treatment options should be considered. Analgesic effects of duloxetine have been documented in OA but is associated with a high risk of side effects [42] and the underlying mechanisms of the analgesic response are widely unknown.

### The underlying mechanisms for the analgesic effect of duloxetine in osteoarthritis

High pain intensity in combination with a low grade of radiological OA seems to apply to a subset of patients with OA who are highly pain sensitive [2, 22]. Emerging evidence suggests that patients with OA and highly pain sensitive might be at higher risk of poor response to exercise-based therapy [27, 40], analgesic effect of NSAIDs [21, 46, 48], and total joint arthroplasty [5, 15, 33, 36, 37, 39, 52, 57–59], which could suggest that patients with OA who are severely centrally pain sensitive do not respond to standard OA treatment.

Preclinical trials have established that serotonin and noradrenaline are important neurotransmitters for descending noxious inhibitory control [7–9, 35]. Duloxetine is a serotonin-noradrenalin re-uptake inhibitor and the analgesic effect may occur through a facilitation of the descending pain inhibitory control system. The human surrogate model for descending noxious inhibitory control as assessed in preclinical studies is CPM [60], although the indirect measurements that can be done in humans can only be considered a proxy for the direct electrophysiological recordings [19]. This trial will assess CPM before and after administration of duloxetine for 18 weeks together with measures of central sensitization (PPTs, TSP) and will thereby be able to demonstrate if the sensitized pain system is modifiable by duloxetine and possibly link these mechanisms to the analgesic effects.

### Patient adherence to protocol

The current study applies a stand-alone intervention of duloxetine or placebo and allows patients to use up to 4000 mg paracetamol daily but excludes the use of NSAIDs. This might challenge the patient adherence to the protocol and the enrollment in the study. In addition, several known side-effects are associated with the use of duloxetine in OA [42] and this can further challenge the adherence to the protocol.

Finally, the study recruitment and participant adherence are limited due to the outbreak of SARS-CoV-2 [29]. Originally, this study planned to enroll 40 patients for a 14-week intervention period, but due to the pandemic the intervention period was extended to 18 weeks (protocol version 6, 3 June 2020) to ensure participant adherence to the protocol.

### Clinical implications

Personalized medicine is focused on providing the right treatment to the right patient. This has not been explored in-depth in patients with chronic pain but evidence suggests that e.g. patients with painful diabetic neuropathies and impaired CPM might benefit from duloxetine [61]. Similarly, a study found that ketamine can reduce TSP in patients with fibromyalgia [25]. Finally, TSP have been used as a marker to identify patients with chronic pancreatitis responding to gabapentinoids [41]. These studies indicate a link between QST and responses to pharmacological interventions and the current study will further demonstrate if this link exists in patients with OA.

## Trial status

The trial is ongoing and is currently recruiting patients. The first patient was enrolled on January 24, 2020, and the last visit of the last patient is expected to take place end of November 2022. This document is based on protocol version 6 of 3 June 2020. The trail was registered with ClinicalTrails.gov (NCT04224584, link: https://www.clinicaltrials.gov/ct2/show/NCT04224584?term=NCT04224584&draw=2&rank=1) on 6 January 2020 and EudraCT (number: 2019-003437-42) on 22 October 2019.

## Data Availability

The data used and analyzed during the trial will be available by contacting the corresponding author on reasonable request. The sponsor will have full access to the completely patient-anonymized dataset.

## Abbreviations

ACR: American College of Rheumatology
cPDT: Cuff pain detection threshold
cPTT: Cuff pain tolerance threshold
OA: Osteoarthritis
OARSI: Osteoarthritis Research Society International
NSAIDs: Nonsteroidal anti-inflammatory drugs
PPT: Pressure pain threshold
TSP: Temporal summation of pain
CPM: Conditioned pain modulation
